# Prevalence and Treatment of Osteoporosis Prior to Elective Shoulder Arthroplasty

**DOI:** 10.5435/JAAOSGlobal-D-20-00204

**Published:** 2020-12-07

**Authors:** James T. Bernatz, Andrew E. Brooks, Benjamin P. Nguyen, Edward D. Shin, Neil C. Binkley, Paul A. Anderson, Brian F. Grogan

**Affiliations:** From the Department of Orthopedics & Rehabilitation (Dr. Bernatz, Dr. Brooks, Mr. Nguyen, Mr. Shin, Dr. Anderson, and Dr. Grogan), and the Divisions of Endocrinology and Geriatrics, Department of Medicine (Dr. Binkley), University of Wisconsin School of Medicine and Public Health, Madison.

## Abstract

**Introduction:**

The rate of preoperative osteoporosis in lower extremity arthroplasty is 33%. The prevalence of osteoporosis in shoulder arthroplasty patients is inadequately studied. The purpose of this study was to (1) determine the prevalence of osteoporosis in patients undergoing elective shoulder arthroplasty, (2) report the percentage of patients having dual-energy x-ray absorptiometry (DEXA) testing before surgery, and (3) determine the percentage of patients who have been prescribed osteoporosis medications within 6 months before or after surgery.

**Methods:**

This retrospective case series included all adults aged 50 years and older who underwent elective shoulder arthroplasty at a single tertiary care center over an 8-year period. National Osteoporosis Foundation (NOF) criteria for screening and treatment were applied.

**Results:**

Two hundred fifty-one patients met the inclusion criteria; 171 (68%) met the criteria for DEXA testing, but only 31 (12%) had this testing within 2 years preoperatively. Eighty patients (32%) met the NOF criteria for receipt of pharmacologic osteoporosis treatment, and 17/80 (21%) received a prescription for pharmacotherapy.

**Discussion:**

Two-thirds of elective shoulder arthroplasty patients meet the criteria to have bone mineral density measurement done, but less than 20% have this done. One in three elective shoulder arthroplasty patients meet the criteria to receive osteoporosis medications, but only 20% of these patients receive therapy.

Over 100,000 shoulder arthroplasties are done in the United States annually, with the number increasing in recent years.^1^ As the population ages, the prevalence of osteoporosis is expected to grow.^2^ The effect of osteoporosis on outcomes in shoulder arthroplasty is not well studied, but higher rates of periprosthetic fracture and need for revision surgery in patients with osteoporosis compared with those without osteoporosis have been reported.^3^

Osteoporosis in patients undergoing other major joint arthroplasty (ie, hip and knee) is correlated with worse outcomes such as aseptic loosening, component malpositioning, and subsidence.^4,5,6,7^ Moreover, osteoporosis before hip and knee arthroplasties is common; it is present in approximately one-third of patients but is under-recognized and undertreated.^8-10^ A recent study found that most arthroplasty patients met guidelines for bone mineral density (BMD) screening, yet most did not have this done before surgery.^8^ Furthermore, BMD in the ipsilateral femur has been shown to decrease by 15% after total knee arthroplasty, suggesting that the arthroplasty surgery or implants have a lasting effect on periprosthetic bone.^11^ To mitigate the potential complications of osteoporosis on arthroplasty, the scope of bone health disorders in this population must first be understood.

Osteoporosis is widely diagnosed using the World Health Organization criteria based on dual-energy x-ray absorptiometry (DEXA) scanning, with osteoporosis defined as a BMD T-score less than or equal to −2.5 and osteopenia between −1 and −2.5. However, others recommend osteoporosis be diagnosed based on fragility fractures or fracture risk estimates using the Fracture Risk Assessment Tool (FRAX).^12,13^

We hypothesize that preoperative osteoporosis is common, under-recognized, and undertreated before elective shoulder arthroplasty. We did not seek to measure outcomes in this study but rather to extend the principles of the American Orthopaedic Association's Own The Bone campaign to a high-risk elective surgery population. The purpose of this study was to (1) determine the prevalence of osteoporosis (using updated clinical diagnosis guidelines) in patients undergoing elective shoulder arthroplasty at a tertiary care center, (2) report the percentage of patients having DEXA testing before surgery, and (3) determine the percentage of patients who have been prescribed osteoporosis medications within 6 months before or after surgery.

## Methods

We conducted a retrospective review of patients undergoing shoulder arthroplasty (anatomic total shoulder arthroplasty [aTSA], reverse total shoulder arthroplasty [rTSA], and hemiarthroplasty) at a single tertiary-care center over an 8-year period from January 1, 2011, to January 1, 2019. The study was granted exemption by the Institutional Review Board under 45 CFR 46.102(d). Patients were identified by the Current Procedural Terminology codes for shoulder arthroplasty (CPT 23470 and 23472-4). Inclusion criteria were any patient older than 50 years of age undergoing one of the above arthroplasty procedures. Exclusion criteria were patients with acute fracture (eg, hemiarthroplasty for acute proximal humerus fracture) or revision surgery for infection. If a patient had multiple shoulder arthroplasties during the study period, only the first surgery was included in this analysis. Type of arthroplasty and indication for surgery was recorded from the surgeons' operative report. Ten 10 surgeons were included with the following fellowship training: 4 hand/upper extremity, 2 shoulder/elbow surgeons, 2 nonfellowship-trained surgeons, 1 sports medicine surgeon, and 1 orthopaedic oncology surgeon.

Electronic medical records (EMRs) were reviewed for demographic information, preoperative osteoporosis risk factors (Table 1), previous DEXA testing, and osteoporosis pharmacotherapy (prescription within 6 months before or after surgery). The expanded Care Everywhere Network of the EMR was also reviewed to include available electronic data from participating outside medical facilities. DEXA was assessed by the lead author for accuracy before extraction of BMD data and T-scores. Inaccurate DEXA results because of improper default identification of bone edges and regions of interest were excluded. The lowest T-score from either the average of 2+ lumbar vertebrae (excluding vertebrae with degenerative or surgical changes) or proximal femur (femoral neck and total femur) was recorded. The National Osteoporosis Foundation (NOF), National Bone Health Alliance, and the United States Preventive Services Task Force criteria for BMD testing (Table 2) and pharmacologic osteoporosis treatment (Table 3) were applied to all patients.^12,13^ The term “appropriately screened” was used to describe patients for whom BMD testing was indicated and who had undergone DEXA in the 2 years before surgery. The term “appropriately treated” was used to describe patients for whom treatment was indicated (Table 3) and who received a prescription for antiosteoporosis pharmacotherapy within 6 months before or after surgery. Previous osteoporosis medications included bisphosphonates, denosumab, raloxifene, abaloparatide, or teriparatide. The FRAX calculator was used to estimate fracture risk without BMD for all patients. A separate calculation of FRAX with BMD was completed for those who had DEXA testing in the 2 years preoperatively.

**Table 1:** Clinical Risk Factors Included in the Fracture Risk Assessment Tool^12^

1. Age
2. Sex
3. Body mass index
4. Previous fracture^a^
5. Parent fractured hip
6. Current smoking
7. Glucocorticoid use^b^
8. Rheumatoid arthritis
9. Secondary osteoporosis^c^
10. Alcohol three or more units per day
11. Femoral neck bone mineral density, when available (g/cm^2^)

a Previous fracture in adult life occurring after low-energy trauma.

b Equivalent to 5 mg prednisolone daily currently or for >3 months in the past.

c Secondary cause of osteoporosis: type 1 diabetes, osteogenesis imperfecta, untreated long-standing hyperthyroidism, hypogonadism or premature menopause, chronic malnutrition, or malabsorption and chronic liver disease.

**Table 2:** NOF and USPSTF Guidelines for BMD Screening^12,13^

Women	Men
All age ≥65	All age ≥70
Younger postmenopausal and women in the menopausal transition with clinical risk factors for fracture^a^	Age 50-69 with clinical risk factors for fracture^a^
Age ≥50 who have had an adult age fracture	
FRAX MOF risk without knowledge of BMD is ≥8.4%	

BMD = bone mineral density, FRAX = Fracture Risk Assessment Tool, MOF = major osteoporotic fracture, NOF = National Osteoporosis Foundation, USPSTF = United States Preventive Services Task Force

a Clinical risk factors found in Table 1.

**Table 3:** WHO, NOF, and NBHA NOF Guidelines for Pharmacologic Treatment of Osteoporosis^12,13^

T-score ≤2.5 at the femoral neck or spine^a^
History of hip or vertebral fracture
T-score between −1 and −2.5 at the femoral neck or spine and a 10-year risk of hip fracture ≥3% or major osteoporotic fracture ≥20%

NBHA = National Bone Health Alliance, NOF = National Osteoporosis Foundation, WHO = World Health Organization

a After appropriate evaluation to exclude secondary causes.

Statistical analysis was completed using Microsoft Excel. Continuous variables were compared using 2-sample *t* tests assuming unequal variance. *P* values less than 0.05 were considered statistically significant. An a priori power analysis was not completed because the aim of the study was not to detect a difference between groups. In some areas, descriptive statistics were used because the intention was not to detect differences between groups but rather to profile the bone health of all patients presenting for shoulder arthroplasty.

No outside funding was involved in this project.

## Results

### Demographics and Indications for Arthroplasty

A total of 263 shoulder arthroplasties were done during the study period. Five were excluded for age younger than 50 years, and seven were excluded for acute fracture. A total of 251 patients (127 F, 124 M) were included in the analysis (Table 4). The mean age was 68 years for all arthroplasty patients (range 50 to 88). The average age by procedure was 61 years for aTSA (range 50 to 88), 71 years for rTSA (range 53 to 88), and 62 years for hemiarthroplasty (range 50 to 85). Ninety-three percent were Caucasian. The most common indication for aTSA and hemiarthroplasty was primary osteoarthritis (93% and 76%, respectively). The most common indication for rTSA was rotator cuff tear arthropathy (57%).

**Table 4:** Demographics and Indications for Shoulder Arthroplasty for all Subjects

Variable	aTSA (n = 126)	rTSA (n = 104)	Hemiarthroplasty (n = 21)	Total (n = 251)
Age (average, range)	61, 50-88	71, 53-88	62, 50-85	68, 50-88
<65	40	27	15	82
65-80	80	67	5	152
>80	6	10	1	17
Sex				
Female	55 (44%)	61 (59%)	11 (52%)	127 (51%)
Ethnicity				
Caucasian	119	97	18	234
African-American	7	6	2	15
Hispanic	0	1	1	2
Preoperative diagnosis				
Primary osteoarthritis	117	38	16	171
Cuff tear arthropathy	3	59	1	63
Post-traumatic	1	6	2	9
Rheumatoid arthritis	4	2	2	8
Revision (aseptic loosening)	—	2	—	2
Osteonecrosis	1	—	—	1

aTSA = anatomic total shoulder arthroplasty, rTSA = reverse total shoulder arthroplasty

### Osteoporosis Screening

**Figure 1:**
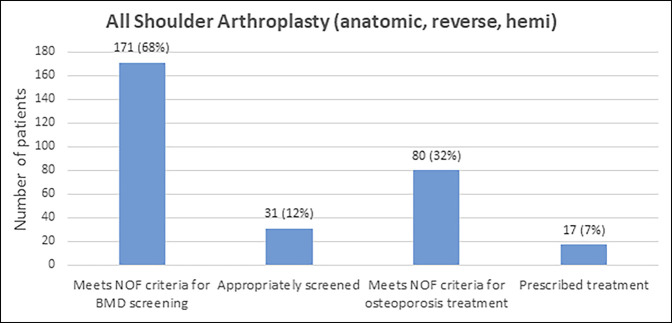
Figure demonstrating that the number of patients meeting the NOF criteria for screening and treatment is greater than those who received screening and treatment. NOF = National Osteoporosis Foundation.

In total, 171 of 251 (68%) patients met the NOF criteria for BMD screening (Table 5, Figure 1), whereas only 31 of 251 patients (12%) had DEXA screening in the 2 years before surgery. Of the 31 patients with DEXA screening in the 2 years before surgery, eight were osteoporotic (T-score ≤2.5), 12 were osteopenic (T-score between −1 and −2.5), and 11 had normal BMD (T-score ≥−1).

**Table 5:** Number of Patients Meeting the Criteria for Bone Health Screening

Criteria for Screening	aTSA (n = 126)	rTSA (n = 104)	Hemiarthroplasty (n = 21)	Total (n = 251)
Age (women >65, men >70)	1	7	1	9
History of low-energy fracture after age 50	8	12	4	24
Age >50 with clinical risk factors for fracture^a^	3	4	0	7
FRAX MOF (without BMD) ≥8.4%	8	6	1	15
Multiple criteria met	60	52	4	116
Total indicated for screening	80	81	10	171
Screened within 2 yrs before surgery	14 (18%)	17 (21%)	0 (0%)	31 (18%)

aTSA = anatomic total shoulder arthroplasty, BMD = bone mineral density, FRAX = Fracture Risk Assessment Tool, MOF = major osteoporotic fracture, rTSA = reverse total shoulder arthroplasty

a Clinical risk factors listed in Table 1.

The lowest T-score average was −1.4, −1.3, and −1.0 for hemiarthroplasty, rTSA, and aTSA, respectively. A total of 24 of the 251 patients had a previous fracture after age of 50. Thus, despite obtaining BMD measurement in only 12%, 29 of these patients had clinical osteoporosis defined as a T-score of ≤−2.5 or a previous fracture.

### Fracture Risk Assessment

**Figure 2:**
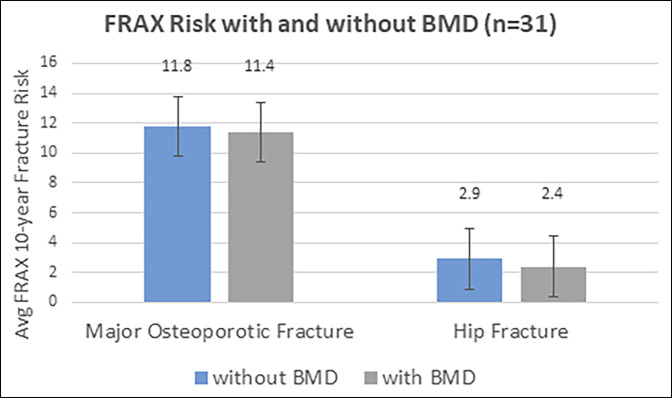
Figure demonstrating the difference between MOF and hip fracture risks when BMD was and was not included in the FRAX calculation. *P* = 0.358 and 0.101 for MOF and hip fracture, respectively. Error bars represent SD. BMD = bone mineral density, FRAX = Fracture Risk Assessment Tool, MOF = major osteoporotic fracture.

For all patients, the median calculated 10-year fracture risk without knowledge of BMD was 1.7% (SD = 3.5) for hip fracture and 8.8% (7.0) for major osteoporotic fracture (MOF) (range 0.1% to 27% and 2.2% to 45% for hip and MOF, respectively). Twenty patients (8%) had a 10-year MOF risk >20%, and 57 patients (22%) had a 10-year hip fracture risk >3%. No statistically significant difference was noted in fracture risk if BMD was or was not included in the FRAX calculation. Average MOF risk was 11.8% without BMD and 11.4% with BMD (*P* = 0.358), whereas hip fracture risk was 2.9% without BMD and 2.4% with BMD (*P* = 0.101) (Figure 2).

### Osteoporosis Treatment

Overall, 80 of 251 patients (32%) met the NOF criteria for osteoporosis treatment (Table 6). Twenty-four patients met multiple treatment criteria. For patients only meeting one treatment criteria, treatment indications included T-score between −1 and −2.5 and a 10-year risk of hip fracture greater than 3% or MOF risk greater than 20% (n = 22), history of previous low energy fracture (n = 11), and T-score ≤2.5 at the femoral neck or spine (n = 10).

**Table 6:** Number of Patients Receiving Indicated Treatment for Antiosteoporosis Medication

Criteria for Treatment	aTSA (n = 126)	rTSA (n = 104)	Hemiarthroplasty (n = 21)	Total (n = 251)
Previous low-energy fracture after age 50	4	6	1	11
BMD T-score <−2.5	4	5	1	10
T-score −1 to −2.5 with FRAX hip >3% or MOF >20%	10	12	0	22
Multiple criteria met	17	15	5	37
Total indicated for treatment	35	38	7	80
Received treatment	6 (17%)	10 (26%)	1 (14%)	17 (21%)

BMD = bone mineral density, FRAX = Fracture Risk Assessment Tool, MOF = major osteoporotic fracture

Seventeen of the total 251 patients (7%) had been prescribed osteoporosis pharmacotherapy in the 6 months before or after surgery.

### Case Example

A 72-year-old female with a history of previous hip fracture underwent aTSA for primary glenohumeral osteoarthritis. Her last DEXA testing was 7 years before, at which time her femoral neck T-score was −2.2, and she was not prescribed any osteoporosis pharmacotherapy. One year postoperatively, she had a mechanical ground level fall and sustained a periprosthetic proximal humerus fracture. At that time, she was referred to the fracture liaison service, and she was found to have a T-score −2.9 at the femoral neck and a trabecular bone score of 1.141, consistent with degraded bone microarchitecture. Adjusted for trabecular bone score, her FRAX risk was calculated to be 9.2% and 28% for hip and MOF, respectively. She was not found to have any other modifiable risk factors for osteoporosis and was prescribed calcium, vitamin D, and teriparatide.

## Discussion

This study found osteoporosis to be common, underdiagnosed, and undertreated before elective shoulder arthroplasty. In our sample, 68% the met criteria for BMD screening but only 18% of those patients had DEXA testing in the 2 years before surgery. These data suggest that approximately four of five shoulder arthroplasty patients should receive DEXA testing, but this is rarely done. Furthermore, even with a small minority of these patients having BMD measured, 32% of patients met the NOF criteria for osteoporosis treatment. However, only 17 (7%) received a prescription for antiosteoporosis medications within 6 months of surgery.

In the total hip and total knee arthroplasty patients, osteoporosis is associated with intraoperative fractures,^14,15^ periprosthetic fractures,^16,17,18,19^ altered component positioning,^7^ delayed osteointegration,^4^ subsidence,^20^ and aseptic loosening.^5,6^ In TSA, Casp et al^3^ found that patients with osteoporosis had significantly higher rates of periprosthetic fracture and revision surgery after both aTSA and rTSA as compared with matched controls without osteoporosis. Furthermore, Otto et al^21^ found that osteoporosis is a significant risk factor for scapular fractures after rTSA. It remains unknown whether preoperative screening and bone health optimization would be effective in mitigating some of these risks in shoulder arthroplasty. However, we feel that detailing the prevalence of osteoporosis in this patient population and highlighting our current deficiencies in screening and treatment are the first steps in improving metabolic bone care in this at-risk population.

If one defines osteoporosis as elevated fracture risk,^22^ our study found an overall higher prevalence of osteoporosis before shoulder arthroplasty than the study by Casp et al (32% versus 14% to 26%).^3^ This may be due to differences in the patient population; however, we posit that this is because of more inclusive diagnostic criteria. Administrative databases used in the Casp et al study only capture those with a documented diagnosis of osteoporosis, whereas our study evaluated each patient based on previous DEXA screening, clinical history, and FRAX risk calculation. We advocate for the use of this more inclusive criteria for diagnosing and treating osteoporosis. Furthermore, our study intentionally excluded patients undergoing arthroplasty for acute proximal humerus fracture. This potentially excludes patients with low BMD that have sustained a typical low-energy proximal humerus fracture; thus, our data may under-represent the true prevalence of osteoporosis in all-comers undergoing shoulder arthroplasty.

Preoperative CT is often done in shoulder arthroplasty patients to determine glenoid bone stock, measure version and inclination, and plan implant selection.^23^ These data can also be used to “opportunistically” screen for osteoporosis.^24^ Nappo et al^25^ correlated BMD and glenoid neck CT-derived Hounsfield units (HUs). They concluded that a patient with glenoid neck HU below 197 has a 97% chance of having low BMD, whereas a patient with HU over 257 likely has normal BMD. Similarly, Pervaiz et al examined CT data from the proximal humerus and found that patients with osteoporosis had an average proximal humerus HU of 121.3, whereas those with osteoporosis had an HU of 92.1.^26^ This opportunistic screening at no additional cost, radiation, or time may help identify patients who would benefit from further preoperative workup and potentially treatment for osteoporosis.

Knowledge of bone status before surgery is of particular importance in arthroplasty because it may affect the decision of whether to use cement and/or a stemmed humeral component. In a survey of 435 orthopaedic surgeons, Maier et al^27^ found that over 60% of surgeons indicated low BMD would be a reason to reconsider operation strategies (in hip arthroplasty); however, only 4% done BMD measurement preoperatively. In shoulder arthroplasty, specifically, bone quality may alter a surgeon's choice of implant and surgical technique. Osteoporosis has been described as a relative contraindication to the use of a stemless humeral prosthesis in aTSA.^28^ Surgeons may also elect to cement a stemmed humeral component in the setting of poor humeral bone quality to avoid the risk of intraoperative fracture associated with press-fitting a noncemented component.

Several studies suggest improved outcomes in lower extremity arthroplasty and spine fusions when osteoporosis is treated perioperatively. A meta-analysis of four studies found that long-term diphosphonate use correlates with reduced revision rates after total hip arthroplasty and total knee arthroplasty (relative risk 0.48).^29^ In thoracolumbar spine fusion, a meta-analysis found that teriparatide use is associated with higher fusion rates than untreated controls (odds ratio 2.3, *P* < 0.001).^30^ To our knowledge, no studies have been found on osteoporosis treatment and shoulder arthroplasty. With no clear published guidelines for this subset of orthopaedic patients, the authors choose to follow the NOF recommendations for osteoporosis treatment (Table 6).^12^ Osteoporosis treatment with bisphosphonates does carry noteworthy risks including gastrointestinal inflammation, osteonecrosis of the jaw, and atypical femur fractures; however, these are rare and more commonly encountered with high-dose IV treatments for patients with cancer.^12^ Anabolic agents (eg, teriparatide) are better tolerated and have side effects including leg cramps, nausea, and dizziness. Further research is needed to identify the optimum indications for treatment, medication to use, and timing of such treatment.

This study is limited by its use of patients from a single institution and geographic area. The prevalence of osteoporosis and screening/treatment practices may not be generalizable to other populations. The population included in this study had an average age of 68 years, with aTSA patients averaging 61 years. Therefore, the osteoporosis prevalence of an older population may be even higher. We excluded those with proximal humerus fractures requiring arthroplasty, which almost always would be associated with osteoporosis. This study is not powered nor designed to show causality between osteoporosis and poor outcomes. Rather, we report a high prevalence of osteoporosis, which in other studies of major joint arthroplasty, has been correlated to adverse outcomes.^1,4,7,14-16,18-20^ The EMR may have missing data. We queried the expanded Care Everywhere Network as well to decrease missing data, but history and medications recorded elsewhere (ie, another state) were not captured. In addition, family history of hip fracture is a component of the FRAX calculation that is not well recorded in the EMR but can affect the fracture risk. This study was underpowered to detect a difference in FRAX risk calculated with and without BMD.

We find osteoporosis to be common, underdiagnosed, and undertreated before elective shoulder arthroplasty. Easy-to-follow clinical guidelines and already-available CT data can potentially help identify patients who should be screened with DEXA. We think that bone health screening and optimization should be considered before elective shoulder arthroplasty. Further research is needed to examine the outcomes of shoulder arthroplasty in patients with osteoporosis, the cost-effectiveness of osteoporosis screening, and the optimum use and timing of osteoporosis treatment in the perioperative and postoperative periods.
